# Non-dominant hand movement facilitates the frontal N30 somatosensory evoked potential

**DOI:** 10.1186/1471-2202-11-112

**Published:** 2010-09-07

**Authors:** Wynn Legon, Jennifer K Dionne, Sean K Meehan, W Richard Staines

**Affiliations:** 1 Department of Kinesiology, University of Waterloo, 200 University Ave. West, Waterloo, Ontario, N2L 3G1, Canada

## Abstract

**Background:**

Previous literature has shown that the frontal N30 is increased during movement of the hand contralateral to median nerve stimulation. This finding was a result of non-dominant left hand movement in right-handed participants. It is unclear however if the effect depends upon non-dominant hand movement or if this is a generalized phenomenon across the upper-limbs. This study tests the effect of dominant and non-dominant hand movement upon contralateral frontal and parietal somatosensory evoked potentials (SEPs) and further tests if this relationship persists in left hand dominant participants. Median nerve SEPs were elicited from the wrist contralateral to movement in both right hand and left hand dominant participants alternating the movement hand in separate blocks. Participants were required to volitionally squeeze (~ 20% of a maximal voluntary contraction) a pressure-sensitive bulb every ~3 seconds with the hand contralateral to median nerve stimulation. SEPs were continuously collected during the task and individual traces were grouped into time bins relative to movement according to the timing of components of the Bereitschaftspotential. SEPs were then averaged and quantified from both FCZ and CP3/4 scalp electrode sites during both the squeeze task and at rest.

**Results:**

The N30 is facilitated during non-dominant hand movement in both right and left hand dominant individuals. There was no effect for dominant hand movement in either group.

**Conclusions:**

N30 amplitude increase may be a result of altered sensory gating from motor areas known to be specifically active during non-dominant hand movement.

## Background

Somatosensory information from the hand is first processed cortically in contralateral primary somatosensory cortex (S1) but also reaches classically defined motor areas in the frontal cortex [1]. The N30 component of the median nerve somatosensory evoked potential (SEP) is a promising physiological index of somatosensory inflow to frontal motor cortical structures. It has been hypothesized to be generated in the supplementary motor area (SMA) [2,3] and its amplitude to reflect incoming proprioceptive sensory information [4-6].

The N30 has been investigated under various sensory-motor paradigms and generally been shown to behave similarly to parietal SEP components [7-9] though does display unique modulation independent of parietal SEP components under specific motor-related conditions such as mental imagery and ideation [10,11] as well as a distinct attenuation in Parkinson's disease (PD). The depressed N30 in PD patients can be transiently facilitated with dopamine agonist administration [12,13], pallidotomy [14] or globus pallidus interal segment (GPi)/sub-thalamic nucleus (STN) stimulation [15] and as such, N30 amplitude has been hypothesized to reflect the proper functioning of specific motor pathways linking basal ganglia to frontal cortex [16].

The N30 has previously been demonstrated to be facilitated independently of parietal components during upper-limb movements contralateral to the stimulating site [17,18]. For example, Legon et al. [18] demonstrated that N30 facilitation only occurs during but not before or after voluntary movement, suggesting an influence of motor cortical activity as a result of contralateral hand movement. However, it is unclear if N30 facilitation is contingent upon the relationship between the side of sensory input and motor output as a reversal of sensory input and motor output across the upper limbs was not investigated. It may be that hemispheric dominance affects sensorimotor integration across the upper-limbs as the effect of sensory input upon motor cortical activity is different across hemispheres [19]. Furthermore, use of the non-dominant hand results in unique recruitment of basal-ganglia nuclei [20], SMA [21], ipsilateral motor cortex [22,23] and subsequent differences in inter-hemispheric inhibition between motor cortices both before and during movement [24,25].

There are a few reports employing SEPs that have shown an effect of contralateral upper-limb movement upon SEP amplitudes [26,27]. Hoshiyama & Kakigi [26] had both right and left-hand dominant participants perform a tracing task with either their dominant or non-dominant hand while recording SEPs from the hand contralateral to movement. Interestingly, non-dominant hand use resulted in an attenuation of N30 amplitude; a result at odds with the work of both Rossini et al. [17] and Legon et al. [18]. This discrepancy may be a result of increased demands associated with the tracing task whereas a simple volitional movement was performed in the former studies. Despite this, modulation of the N30 in the Hoshiyama & Kakigi [26] study only occurred for tracing performed with the non-dominant hand in both right and left hand dominant participants suggesting a specific relationship for N30 modulation during non-dominant upper-limb motor output regardless of hand dominance.

These results would suggest a link between the N30 and non-dominant hand use but it is nonetheless unclear if handedness has an effect upon the integration of sensory input and motor output across the upper-limbs and if this translates to modulation of either parietal or frontal SEP components during a simple volitional motor task that is not highly skilled or requires vision. Hoshiyama & Kakigi [26] reported no differences between right and left hand dominant individuals but anatomical [28] and cortical excitability differences between left and right hand dominant individuals have been reported [29-35] and may contribute to N30 amplitude modulation.

It is the purpose of the current study to determine if N30 facilitation observed by Legon et al. [18] is exclusive to movement of the non-dominant upper-limb and further if this relationship persists in left hand dominant individuals. Participants were instructed to voluntarily squeeze a pressure-sensitive bulb roughly every 3 seconds with either their dominant or non-dominant hand while median nerve stimulations were continuously delivered to the contralateral wrist. These stimulations were later binned according to timings of the Bereitschaftspotential to assess amplitude differences of the N30 before, during and after movement of the contralateral hand. It is hypothesized that N30 facilitation is specific to movement of the non-dominant hand in both right and left hand dominant individuals due to differences in cortical activation during non-dominant hand movement or potentially through differences in centrifugal gating of peripheral sensory inputs between the limbs.

## Results

All eight left-handed and right-handed participants showed clear frontal and parietal SEPs. No latency differences were observed for any of the SEPs measured and M-wave amplitudes (an electromyographic (EMG) wave resulting from the direct stimulation of the motoneuronal axons serving the thenar musculature) displayed no differences across conditions.

### Frontal N30

The three-way mixed ANOVA with between subjects factor HANDEDNESS (Right Hand dominant; Left Hand dominant) and within subjects' factors MOVEMENT HAND (Dominant; Non-dominant) and TIMING relative to movement (Early Bereitschaftspotential (EBP); Late Bereitschaftspotential (LBP); Movement (MVMT); Post-Movement (PMVMT)) revealed a between subjects effect of HANDEDNESS (F (1, 14) = 4.39, p = 0.05), a main effect of TIMING (F (3, 42) = 4.16, p = 0.01), and an interaction of MOVEMENT HAND × TIMING (F (3, 42) = 3.76, p = 0.02). The between subjects effect was driven by a larger N30 amplitude as a whole for the left-handed group collapsed across movement hand and timing epochs relative to control (116% vs. 102% (t (126) = 1.99, = 0.05)). The interaction was investigated with one-way repeated measures ANOVAs with factor TIMING for each movement hand in left hand dominant and right hand dominant groups.

### Left Hand Dominant

**Figure 1:**
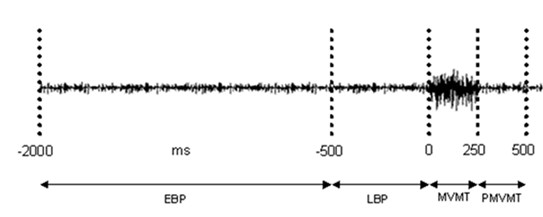
**Timing epochs used to capture SEPs relative to movement**. Example of raw EMG from flexor digitorum superficialis of the hand performing the voluntary squeeze. Timing windows used to divide median nerve stimulations into respective epochs relative to the onset (0 ms) of EMG are shown. (EBP) Early Bereitschaftspotential (-2000 ms to -500 ms); (LBP) Late Bereitschaftspotential (-500 ms to -1 ms); (MVMT) Movement (0 ms to +250 ms); (PMVMT) Post-Movement (+251 ms to +500 ms).

There was no effect of TIMING associated with dominant hand movement in the left hand dominant group (F (3, 21) = 0.38, p = 0.77) whereas there was an effect of TIMING associated with non-dominant hand movement (F (3, 21) = 5.90, p = 0.004). Contrasts revealed N30 amplitude to be larger during the MVMT epoch as compared to the EBP epoch (p < 0.05), LBP epoch (p < 0.05) and PMVMT epoch (p < 0.05) (see Figure 1, 2 &3; Table 1).

**Figure 2:**
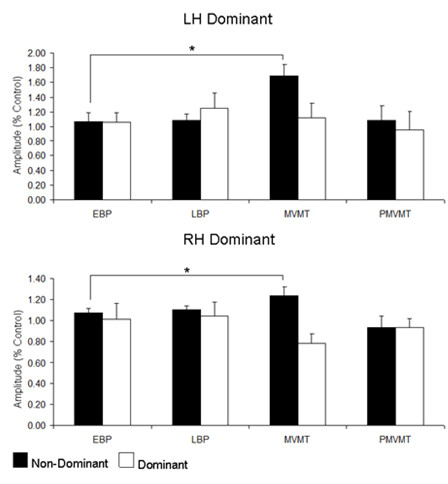
**Group Average N30 amplitudes**. Group average (n = 8) bar graphs for left hand dominant (top) and right hand dominant (bottom) groups for each of the timing epochs. Early Bereitschaftspotential (EBP); Late Bereitschaftspotential (LBP); Movement (MVMT); Post-Movement (PMVMT). White bars indicate movement performed with the dominant hand. Black bars indicate movement performed with the non-dominant hand. Values are expressed relative to control values. Error bars are ± SEM. * denotes significance p < 0.05.

**Figure 3:**
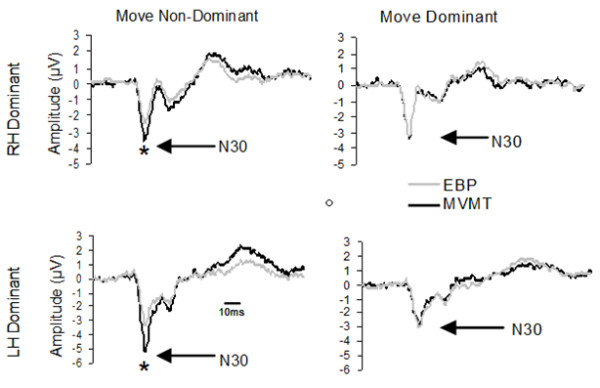
**Group Average FCZ traces**. Group average (n = 8) traces recorded from electrode site FCZ. Top row traces recorded from right hand (RH) dominant group; Bottom row traces recorded from left hand (LH) Dominant group. Left column represents movement performed with the non-dominant hand, right column movement performed with the dominant hand. Light trace a result of median nerve stimulations that fell within the Early Bereitschaftspotential (EBP) epoch; dark trace a result of median nerve stimulations that fell within the Movement epoch (MVMT). * denotes significance p < 0.05.

**Table 1:** EP amplitudes.

Left hand dominant									
**Non-dominant hand movement**					**Dominant hand movement**				

	**FCZ**	**CP3**				**FCZ**	**CP4**		
	**N30**	**N20**	**P27**	**P50**		**N30**	**N20**	**P27**	**P50**

EBP	1.07 (0.12)	1.20 (0.09)	1.09 (0.13)	0.83 (0.11)	EBP	1.06 (0.13)	0.91 (0.16)	0.95 (0.33)	0.89 (0.11)
LBP	1.09 (0.08)	1.44 (0.09)	1.07 (0.14)	1.12 (0.16)	LBP	1.25 (0.21)	1.02 (0.17)	1.12 (0.09)	1.22 (0.24)
MVMT	1.69* (0.15)	1.06 (0.22)	0.95 (0.24)	0.62 (0.09)	MVMT	1.12 (0.20)	0.85 (0.10)	1.04 (0.17)	0.91 (0.22)
PMVMT	1.08 (0.20)	1.28 (0.18)	0.94 (0.15)	0.93 (0.29)	PMVMT	0.95 (0.26)	0.77 (0.10)	1.21 (0.10)	0.88 (0.11)

**Right hand dominant**									

**Non-dominant hand movement**					**Dominant hand movement**				

	**FCZ**	**CP4**				**FCZ**	**CP3**		
	**N30**	**N20**	**P27**	**P50**		**N30**	**P20**	**P27**	**P50**

EBP	1.07 (0.04)	0.97 (0.07)	0.96 (0.06)	1.00 (0.08)	EBP	1.02 (0.15)	1.11 (0.19)	1.06 (0.09)	1.16 (0.29)
LBP	1.10 (0.04)	1.04 (0.07)	0.91 (0.09)	0.94 (0.12)	LBP	1.05 (0.13)	1.19 (0.23)	1.27 (0.15)	1.19 (0.49)
MVMT	1.24* (0.08)	1.15 (0.10)	0.87 (0.08)	0.56 (0.13)	MVMT	0.79 (0.09)	1.01 (0.20)	1.24 (0.21)	1.18 (0.35)
PMVMT	0.93 (0.11)	1.11 (0.14)	0.93 (0.09)	1.13 (0.26)	PMVMT	0.94 (0.08)	1.28 (0.27)	1.32 (0.20)	1.41 (0.47)

Mean (± SEM) of labelled potentials recorded from labelled electrode sites (FCZ, CP3/4) as a result of median nerve stimulation contralateral to hand movement. Top data from left hand dominant group; bottom data from right hand dominant group. (EBP) early Bereitschaftspotential; (LBP) late Bereitschaftspotential; (MVMT) movement; (PMVMT) post movement. All values are expressed relative to control value (1.00). * denotes significance p < 0.05.

### Right Hand Dominant

There was no effect of TIMING associated with dominant hand movement (F (3, 21) = 1.95, p = 0.15). Non-dominant hand movement revealed an effect of TIMING (F (3, 21) = 4.02, p = 0.02). Contrasts revealed N30 amplitude to be significantly greater during the MVMT epoch as compared to the EBP epoch (p < 0.05) and the PMVMT epoch (p < 0.05) (see Figure 1, 2 &3; Table 1).

### Parietal N20

**Figure 4:**
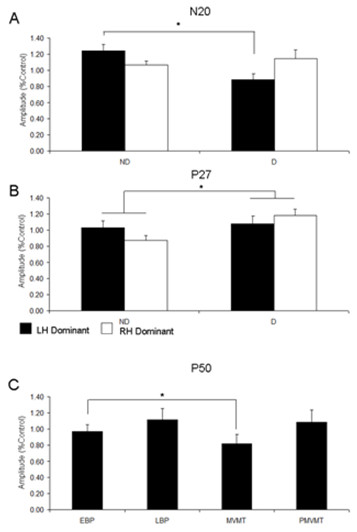
**Group Average Parietal Potentials**. Group average (n = 8) bar graphs for parietal potentials (A) N20, (B) P27 and (C) P50 as recorded from electrode site(s) CP3/4. Amplitudes are expressed relative to control values. For bar graphs A & B, (ND) non-dominant hand movement; (D) dominant hand movement. White bars represent group data from the right hand dominant group; black bars represent group data from the left hand dominant group. P50 amplitudes (C) are collapsed across handedness and represent amplitude for timing epochs Early Bereitschaftspotential (EBP); Late Bereitschaftspotential (LBP); Movement (MVMT); Post-Movement (PMVMT). Error bars are ± SEM. * denotes significance p < 0.05.

The three-way mixed ANOVA revealed an interaction of MOVEMENT HAND × HANDEDNESS (F (1, 14) = 4.50, p = 0.05). N20 amplitude is larger from dominant hand stimulation as compared to non-dominant hand stimulation in left-hand dominant participants (t (31) = 3.44, p = 0.03). There was no difference between stimulation sites for right hand dominant participants (t (31) = -0.81, p = 0.43). As such, stimulation site affects N20 amplitude in left hand dominant participants but not in the right hand dominant group (see Figure 4a; Table 1).

### Parietal P27

The three-way mixed ANOVA revealed a main effect of MOVEMENT HAND (F (1, 14) = 4.47, p = 0.05). The P27 is larger when the dominant hand is the movement hand (t (126) = -2.41, p = 0.02) (see Figure 4b; Table 1).

### Parietal P50

The three-way mixed ANOVA revealed a main effect of TIMING (F (3, 42) = 3.38, p = 0.03). Data from both the left hand dominant and right hand dominant groups were collapsed and a one-way repeated measures ANOVA with factor TIMING was performed (F (3, 93) = 3.61, p = 0.02). Contrasts revealed that the P50 amplitude is significantly decreased during the MVMT epoch compared to the LBP (p < 0.05) and the PMVMT epoch (p < 0.05) (see Figure 4c; Table 1).

## Discussion

It was the purpose of this study to determine the effect of hand movement and hand dominance upon the amplitude of the N30 SEP during a simple volitional movement. Previous literature [17,18] has demonstrated a frontal N30 amplitude increase during voluntary non-dominant hand movement in right hand dominant participants. This study demonstrates a facilitation of frontal N30 SEP amplitude during non-dominant hand movement but not during dominant hand movement and further that this relationship is true for left hand dominant individuals as well. It is currently unclear why N30 amplitude is facilitated only during non-dominant hand movement but may be the result of specific activation of basal ganglia, SMA and/or primary motor cortex for non-dominant as compared to dominant hand use [20-23]. In addition to the understood motor roles of these areas, all receive sensory input [36-39] and thus are candidate areas for the integration of sensory input for motor control. As such, peripheral sensory input from the dominant limb may be differentially modulated as compared to non-dominant inputs during contralateral hand use by altered active centrifugal gating mechanisms.

The N30 has been hypothesized to be the result of peripheral proprioceptive afference [4,5] and its amplitude may reflect the proper functioning of centripetal and/or centrifugal sensory gating mechanisms [6]. The persistent finding of a depressed or absent N30 in the PD population [13,40,41] suggests a link between it and the basal ganglia dopaminergic system, such that the amplitude of the N30 reflects the healthy functioning of the basal-ganglia, cortico-cortical motor loops [16]. The dopaminergic hypothesis for N30 amplitude is further corroborated by the findings that dopaminergic administration [12,13], pallidotomy [14] and GPi or STN stimulation [15] facilitate the N30 that is paralleled by clinical improvement. Interestingly, N30 amplitude increase in the PD population under these interventions is commonly correlated with a reduction in rigidity [42]. As such, it may very well be that restoration of the basal-ganglia dopaminergic system is not the direct cause of N30 facilitation but rather results in less gating of peripheral sensory inputs to cortical motor structures (SMA) through a reduction in rigidity [41] which acts to inhibit sensory inflow similar to the effects of voluntary or passive movement. This hypothesis is corroborated by the results of Pierantozzi et al. [6] who demonstrated atracurium (nicotinic antagonist) administration to increase the N30 not only in PD patients but also in neuroleptic malignant syndrome patients and healthy controls; groups with intact dopaminergic systems.

Alternately, the N30 amplitude difference may be the result of altered sensory gating mechanisms due to known activity differences in the basal ganglia, SMA and M1 during non-dominant hand use. For example, Francois-Brosseau et al. [20] reported reduced blood oxygenation-level dependant (BOLD) response in the left putamen, thalamus and right caudate for self-initiated finger movement of the non-dominant left hand as compared to the same movement performed by the dominant right hand. Babiloni et al [21] reported that non-dominant hand movement results in right SMA activation whereas dominant hand movement results in both right and left SMA activity. The SMA receives dense afferents from the GPi [43] and in turn, projects to primary motor cortex (M1) [44,45]. The connections of the basal ganglia with SMA are excitatory and those of the SMA to M1 are largely inhibitory in nature. Micro-stimulation of the SMA results in inhibitory post-synaptic potentials on pyramidal neurons in M1 [46] and a conditioning stimulus delivered to the SMA reduces the excitability of M1 to a test pulse [47]. PD patients often show decreased activation of the SMA and increased activation of M1 [48-54] in addition to a lack of cortico-cortical inhibition in M1 [55]. Interestingly, an increase in ipsilateral M1 is a persistent finding during non-dominant hand use [23,24,56,57] a phenomenon that does not often occur for dominant hand use [22,24,58]. The purpose of ipsilateral M1 activity is currently unclear though it has been hypothesized to represent inhibition to presumably prevent mirroring of the dominant hand. Using functional MRI, Kobayashi et al. [24] demonstrated increased intra-cortical inhibition of ipsilateral M1 in those that displayed ipsilateral M1 activity and none in those that did not. An alternate or complimentary theory may be that ipsilateral M1 activity during non-dominant hand use serves to reduce the amount of sensory gating exerted upon the dominant limb. No modulation of N30 amplitude was witnessed either before or after non-dominant hand movement or for any timing epoch explored for the dominant hand, further suggesting a role for ipsilateral M1 activity in the modulation of N30 amplitude. It cannot be said with absolute conviction if this is a purposeful mechanism but may be a way of the central nervous system to increase the fidelity of sensory inputs from the dominant limb to aid motor planning and execution of the less apt non-dominant limb. It is well understood that there are manual asymmetries in motor performance between the dominant and non-dominant limb such that behaviour of the non-dominant limb is usually slower, more variable and less accurate [59,60].

Parkinson's patients show altered response to somatosensory inputs [61,62] and have difficulty performing efficient and precise movements when relying upon kinaesthetic sensory feedback but their performance improves for externally-cued or visually-guided motor tasks [63,64], a phenomenon hypothesized to be the result of incorporating alternate sources of sensory input. Interestingly, the above mentioned differences in basal-ganglia activation for non-dominant hand use during volitional tasks disappeared for an externally triggered task [20]. If indeed N30 amplitude is reflective of the proper functioning of a basal-ganglia - SMA - M1 loop in response to kinaesthetic sensory input, hypotheses would suggest either no increase or a decrease in N30 amplitude during non-dominant hand movement under a condition reliant upon vision which is what Hoshiyama & Kakigi [26] found. They reported a reduction in N30 amplitude for non-dominant as compared to dominant hand movement during a visually guided tracing task. The reduced N30 in this case may reflect increased sensory gating of proprioceptive inputs from the dominant limb that would essentially be less informative or reliable than the visual information. Indeed, Bernier et al. [65] have shown that proprioceptive information is suppressed during a mirror reversal task - a task that is heavily reliant upon visual information. This suppression was reflected in an attenuation of the parietal P27 leaving the N30 unaffected. The lack of N30 change may have been a result of an already depressed N30 as a result of movement-related gating (the moving limb and the stimulated limb were the same) or perhaps because the dominant limb was used to perform the movement in this study. The differences in N30 amplitude during tasks that rely upon vision or cueing versus those that are volitional and largely use proprioceptive feedback is supported by the results of Urushihara et al. [66] who demonstrated an increase in N30 amplitude as a result of pre-motor cortical inhibition from low frequency repetitive trans-cranial magnetic stimulation. The pre-motor cortex, as opposed to the SMA, is preferentially activated for externally triggered vs. internally generated movements [67].

None of the parietal potentials measured displayed an interaction of movement hand and timing as the N30 did. If ipsilateral M1 activity during non-dominant hand use is a source of N30 modulation, it would be reasonable to hypothesize an effect upon potentials generated in S1 due to the dense ipsilateral connectivity [68] and interaction [69] between M1 and S1. Interpretation of the results of the reported parietal potentials is not clear. Generally, inhibition of early parietal potentials as a result of movement is limited to the site of movement [70] and does not occur across the upper limbs though modulation of the N20 and P27 has been reported during contralateral hand movement under specific attention requirements [71]. The lack of a specific effect of movement time and movement hand upon the N20 and P27 parietal potentials may be due to differences in the response of S1 and SMA to somatosensory input. S1 is active to passive tactile stimulation but SMA activity is only present for tactile stimulation that is required for a motor output [72,73], thus the N20 and P27 may not be affected by specific motor activity. It should be noted however that the P50 was specifically inhibited during the movement epoch regardless of the hand performing the task, a finding that corroborates and extends the findings of Legon et al. [18] suggesting that parietal potentials generated outside of area 3b/1 can be modulated by contralateral movement. The P50 has been reported to be generated in S1 [74] and may be specifically generated in area 2 as the preceding P27-N35 complex has been postulated to be generated in area 1 [75]. Area 2 has connectivity with both the SMA [43,44], ipsilateral M1 [76] and secondary somatosensory cortex. The connection with secondary somatosensory cortex provides a route of action for modulation of the P50 independently of the N20 and P27. Secondary somatosensory cortex is active bilaterally in response to unilateral stimulation and more importantly displays movement related activity [27,77,78] similar to the cells of SMA.

Finally, it should be noted that recent research [79,80] has attributed N30 amplitude to a phase-locking of the beta/gamma frequency. Under this hypothesis, evoked potentials may not be the result of localized processing or a fixed latency response to a specific stimulus but rather a reset of oscillatory activity, in the case of the N30 in the beta/gamma frequency. Under the oscillatory model of event-related potentials, an increase in amplitude of a specific potential reflects an increase in the influence of an oscillation which is assumed to be related to specific task processing (see [81] for review). Cebolla et al. [80] recently demonstrated movement-related gating of N30 amplitude to disrupt beta/gamma phase-locking providing additional evidence in support of the oscillatory model of event-related potentials. If indeed this model proves correct, the data from this study would suggest that non-dominant hand movement specifically affects the beta/gamma oscillation, which may be an indication of synchronization or co-activation of cortical and sub-cortical networks (basal ganglia - ipsilateral M1 - SMA) that are specifically active during non-dominant hand movement.

## Conclusion

Non-dominant hand use results in different activation of the basal ganglia, SMA and M1 and an increase in amplitude of the N30 compared to dominant hand use. The relationship between these different activation patterns and N30 amplitude is not clear. The specific attenuation of N30 amplitude in PD has lead to investigation of the basal ganglia and dopaminergic contribution to N30 amplitude. It is clear that classically defined motor pathologies have a sensory contribution and the dysfunction of sensory integration may be critical [82]. These sensory gating mechanisms may be different between the upper-limbs depending upon hand use and reflected in the amplitude of the N30.

## Methods

### Participants

Sixteen subjects participated in one of two experiments performed on separate days. Experiment 1 (Right Hand Dominant) studied eight right-handed (4 female, Age 26 ± 4.6 yrs) and Experiment 2 (Left Hand Dominant), eight left-handed participants (2 female, Age 24 ± 2.2 yr). Handedness was assessed by the Revised Waterloo Handedness Inventory. All participants for respective studies scored strongly right or left handed. Participants provided written informed consent to participate in the study. None of the participants reported any history of neurological or musculoskeletal impairments, and all were paid a nominal fee for their participation. The University of Waterloo, Office of Research Ethics approved all experimental procedures.

### Behavioural Task

Tasks outlined below were identical for both the left hand dominant and right hand dominant groups. Participants were seated comfortably in a desk chair, with arms supported upon a table top, in a sound-attenuating booth and instructed to perform a non-maximal (~ 20% of their maximum) squeeze (~1 s) voluntary contraction against a pressure-sensitive bulb held in either their right or left hand while fixating straight ahead. Participants were instructed to initiate squeezes roughly every 3 s but were allowed to perform successive movements at their own pace. Movements that were within 2 s of each other were discarded. Testing blocks lasted 3 minutes, separated by a 1 minute break repeated five times for each hand. The hand performing the movement was alternated between blocks. Motor and rest periods were indicated by an auditory tone.

### Stimulation and Recording

Stimulation and recording details were similar for both left hand and right hand dominant groups. SEPs were derived from electrical stimulation of the median nerve of the hand contralateral to movement. Stimulation employed square wave pulses of 0.2 ms duration (GRASS S88 stimulator with SIU5 stimulus isolation unit; West Warwick, Rhode Island, USA) delivered through a surface bar electrode, with the anode distal, fixed over the median nerve at the wrist. Median nerve stimuli were delivered during task performance at a constant frequency of 2 Hz and at a voltage sufficient to elicit a noticeable thumb twitch and recordable M-wave. Disposable adhesive surface electrodes were placed over thenar musculature to record the M-wave, an electromyographic (EMG) wave resulting from the direct stimulation of the motoneuronal axons serving the thenar musculature. M-wave amplitude, measured peak-to-peak, was used to confirm the consistency of stimulus intensity. Surface EMG was also recorded from flexor digitorum superficialis of the hand performing the squeeze to monitor performance. EMG recordings were amplified (2000X), band-pass filtered (DC-200 Hz), digitized and stored for later analysis, using customized LabVIEW software (National Instruments; Austin, Texas, USA). The onset of the squeeze was evidenced by the onset of flexor digitorum superficialis EMG activity. SEPs were elicited continuously throughout the squeeze blocks.

Electroencephalographic (EEG) data were recorded from 7 electrode sites (FC2, FCZ, FC1, C4, C3, CP4 and CP3), in accordance with the international 10-20 system for electrode placement referenced to the linked mastoids (impedance < 5 kΩ). EEG data were amplified (40000×), filtered (DC-200 Hz) and digitized at 1000 Hz (NeuroScan 4.3; Compumedics; El Paso, Texas, USA), before being stored on a computer for subsequent analysis. SEPs were extracted by averaging epochs time-locked to the median nerve stimulation (-50 to 300 ms). Individual traces were high-pass filtered (2 Hz) and visually inspected for artefacts (i.e. from blinks, eye movements or contraction of scalp musculature). Any contaminated epochs were eliminated before averaging.

### Data Analysis

Median nerve stimulations were averaged in bins time-locked to EMG onset in flexor digitorum superficialis, according to pre-determined movement epochs corresponding to the different known components of the Bereitschaftspotential (BP): Early BP (-2000 ms to -500 ms); Late BP (-500 ms to -1 ms); Movement (0 ms to +250 ms); Post-Movement (+251 ms to +500 ms) (see Figure 1). Median nerve stimulations that did not fall within the pre-determined epochs were averaged and used as control. SEP traces for each time epoch were a result of 180 randomly chosen stimulations. Latencies and amplitudes of the frontal and parietal SEPs were measured from individual participant averages for each movement epoch from the electrode sites that displayed the maximal amplitudes, FCZ and CP3/4, respectively. Latencies were measured from stimulus onset to the peak of each SEP (frontal N30; parietal N20, P27 and P50). Amplitudes of all potentials were measured as peak voltage relative to a pre-stimulus baseline (50 ms). A clearly defined peak was necessary for inclusion.

For all potentials of interest (frontal N30; parietal N20, P27 and P50) a mixed three-way ANOVA was conducted with between factor Handedness (Left hand dominant, Right hand dominant) and within subject factors Movement Hand (Dominant, Non-dominant) and Timing Epoch (Early Bereitschaftspotential/Late Bereitschaftspotential/Movement/Post-Movement). Analysis was performed on normalized amplitude values relative to control. Part of the data has been previously reported (Legon et al. [18]) but is included in the larger ANOVA presented here.

## Authors' contributions

WL conceived of the experiment and was the primary investigator involved in the data collection and analysis as well as drafting of the manuscript. SKM contributed to experimental design, data analysis, manuscript editing and revision. JKD contributed to data analysis, manuscript writing, editing and revision. WRS (senior author) contributed to experimental design, data analysis, manuscript editing and revision. All authors have read and approved the final manuscript.
